# Pressure-Induced Comproportionation in Palladium Trifluoride

**DOI:** 10.1021/acs.inorgchem.5c00465

**Published:** 2025-04-30

**Authors:** Sylwia Olszewska, Sharad Babu Pillai, Deepak Upadhyay, Kinga Zdun, Jakub Drapała, Klemen Motaln, Mirela Dragomir, Matic Lozinšek, Dominik Kurzydłowski

**Affiliations:** † Faculty of Mathematics and Natural Sciences, Cardinal Stefan Wyszynski University in Warsaw, Warsaw 01-938, Poland; ‡ Faculty of Chemistry, Warsaw University of Technology, Warsaw 00-664, Poland; § Jožef Stefan Institute, Ljubljana 1000, Slovenia; ∥ Jožef Stefan International Postgraduate School, Ljubljana 1000, Slovenia

## Abstract

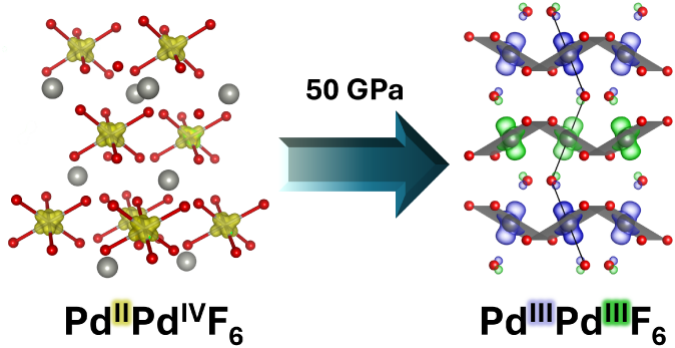

Despite its seeming simple stoichiometry, palladium trifluoride (PdF_3_) is a mixed-valent system better formulated as Pd^II^Pd^IV^F_6_. In an attempt to verify whether the application of high pressure (*P* > 1 GPa) might force this compound to form a genuine palladium(III) fluoride (Pd^III^F_3_), a joint theoretical and experimental study on its properties at large compression was performed. Indeed, hybrid density functional calculations predict the thermodynamic preference for single-valent (comproportionated) polymorphs at pressures exceeding 30 GPa. The ambient-pressure LiSbF_6_-type polymorph of *R*3̅ symmetry was experimentally observed to transform into a triclinic *P*1̅ phase above 42 GPa. While this polymorph is still a mixed-valent compound Pd^II^Pd^IV^F_6_, another phase transition, commencing at ∼50 GPa, introduces a monoclinic *C*2/*c* phase containing genuine Pd^III^ centers. Both high-pressure polymorphs of palladium trifluoride exhibit novel structure types. Moreover, preliminary data suggest that the *C*2/*c* comproportionated structure might host strong one-dimensional antiferromagnetic exchange interactions.

## Abstract 2

Palladium trifluoride is derived from a mixed-valent state (Pd^II^Pd^IV^F_6_) into a phase exhibiting a single palladium valency (Pd^III^F_3_) under high pressure, as shown through a combination of diamond anvil cell experiments (Raman spectroscopy, powder X-ray diffraction) and hybrid DFT calculations.

## Introduction

Transition metal compounds offer a versatile platform for studying crucial chemical and physical phenomena, such as electron transfer, metal-to-insulator transition, charge ordering, spin–spin interactions, etc. On the other hand, oxides and fluorides of these elements are materials of immense technological importance, both in current (optoelectronics, photovoltaics) and future (superconductivity, quantum computing) applications. One of the most important characteristics of transition metal compounds is their ability to exhibit a variety of metal oxidation states (electron counts), sometimes within the same crystal structure leading to mixed-valency (charge ordering).^1,2^ This process competes with possible metallization and structural distortions, and gaining a deeper understanding of the interplay between charge, spin, and structural ordering is a crucial goal for designing new materials with tailored properties. In this context, AB_3_-type compounds are interesting systems due their structural diversity and relation to perovskite (ABX_3_) systems.^3^

Pressure, *P*, as a fundamental thermodynamic variable, can be used to tune the charge-transfer properties in oxides and fluorides.^4−12^ Moreover, novel compounds can form at pressures beyond 1 GPa (10 000 bar). While computations suggest that a number of exotic trifluorides, such as CdF_3_,^13^ HgF_3_,^14^ BaF_3_,^15^ might be attained at high pressure experimental verification of these claims is still missing. In fact, in contrast to the well-researched field of pressure-induced phase transitions in rare-earth trifluorides,^7−9^ experimental data on the high-pressure phase diagrams of transition metal fluorides is still scarce. Only recently, some light has been shed on this topic with diamond anvil cell (DAC) experiments on fourth period trifluorides (FeF_3_,^10,16,17^ and MnF_3_^11^), as well as that of sixth period (AuF_3_).^18^

Herein, a joint computational and experimental study on the high-pressure behavior of palladium trifluoride, a mixed-valent (Pd^II^Pd^IV^F_6_) system which has been proposed to transition into a single-valent Pd^III^F_3_ phase upon compression,^19,20^ is presented.

## Results and Discussion

The synthesis of palladium trifluoride was first reported by Ruff and Ascher in 1929,^21^ and various synthetic paths were reported over the years.^19,20,22−25^ Subsequent powder X-ray diffraction (PXRD) investigations assigned it a VF_3_-type structure (*R*3̅*c* space group) with a single, octahedrally coordinated, Pd site.^26,27^ This suggested a single-valent formulation, in which Pd atoms would exhibit the +3 oxidation state. The existence of Pd^3+^ cations, with their formal 4d^7^ electron count, would lead to an uneven occupation of the *e*_*g*_ orbitals of the octahedral [PdF_6/2_] complex, resulting in a Jahn–Teller-type distortion leading to elongation or contraction of the ideal octahedron. However, such a distortion would not be compatible with the high symmetry of the VF_3_-type structure. Indeed, a subsequent neutron diffraction study showed that the crystal symmetry is lower (*R*3̅ space group, LiSbF_6_-type structure), with two nonequivalent octahedral Pd sites (Wyckoff positions 1*a* and 1*b*) exhibiting markedly different Pd–F distances (2.17 Å for 1*a* and 1.90 Å for 1*b*).^28^ The LiSbF_6_-type geometry clearly indicated a mixed-valent formulation with the 1*a* site occupied by the larger Pd^2+^ cation and the smaller Pd^4+^ ion located on the 1*b* position. The symmetry around both Wyckoff sites is *O*_*h*_, and therefore Pd^2+^ (4d^8^ electron count) should exhibit a high-spin (*S* = 1) configuration, whereas Pd^4+^ (4d^6^) should be diamagnetic (*S* = 0), as in hexafluoropalladates(IV). The existence of such a magnetic arrangement was confirmed by both powder neutron diffraction and magnetic susceptibility measurements.^28,29^ Thus, experimental data clearly indicates that at ambient conditions palladium trifluoride is a mixed-valent compound Pd^II^Pd^IV^F_6_, or in short Pd_2_F_6_, and not Pd^III^F_3_. In fact, compounds containing palladium in the +3 oxidation state are much less common than those exhibiting Pd^II^ and Pd^IV^.^30−32^

### DFT Calculations

It is important to note that DFT modeling performed with the use of the generalized-gradient approximation (GGA), in the form of the Perdew–Burke–Ernzerhof (PBE) functional,^33^ fails to correctly describe this charge ordering and the resultant magnetic properties, as it yields the single-valent VF_3_-type structure (*R*3̅*c*) as the ground state at ambient pressure.^34^ Our PBE calculations confirm this, as we find this polymorph to be 18.6 meV per formula unit (f.u.) lower in energy than the LiSbF_6_-type one. Within the GGA approximation the *R*3̅*c* structure is metallic with small magnetic moment (0.34 μ_B_) distributed equally among all Pd sites (Table 1).

**Table 1:** Properties of the Ground State Structure of Palladium Trifluoride Calculated with the Use of PBE,^33^ r^2^SCAN,^35^ and HSE06 Functionals,^36^ Compared with Experiments^20,28,29^^a^

	S.G.	*a*	α	*V*	Pd_1*a*_–F	Pd_1*b*_–F	*q*_1*a*_	*q*_1*b*_	μ_1*a*_	μ_1*b*_	*E*_*g*_
PBE	*R*3̅*c*	5.60	55.66	111.9	2.03		1.6		0.34		0
r^2^SCAN	*R*3̅	5.53	53.69	102.0	2.10	1.95	1.5	1.8	1.31	0.22	0.6
HSE06	*R*3̅	5.55	54.93	106.4	2.16	1.91	1.5	2.0	1.59	0.07	2.7
Exp.	*R*3̅	5.52(1)	53.90(2)	102.0	2.17	1.90	–	–	1.75	0.00	–

a Cell vector (*a*) for the primitive cell setting and Pd–F distances for 1*a* and 1*b* metal sites (Pd_1*a*_–F and Pd_1*b*_–F) are given in Å, Mulliken charges of Pd atoms (*q*_1*a*_, *q*_1*b*_) in elementary charge (*e*), cell angle (α) in degrees (°), volume (*V*) in Å^3^, magnetic moments (μ_1*a*_, μ_1*b*_) in Bohr magnetons (μ_B_), the electronic band gap (*E*_g_) in eV. For the *R*3̅*c* structure Pd_1*a*_–F = Pd_1*b*_–F, *q*_1*a*_ = *q*_1*b*_, and μ_1*a*_ = μ_1*b*_.

In order to attain a better theoretical description of Pd_2_F_6_, its properties were modeled with the more advanced r^2^SCAN meta-GGA functional.^35^ This approach reduces the difference between experiment and theory yielding the correct *R*3̅ structure as the ground state, with lattice constants very close to the experimental ones (Table 1). This result is in line with previous findings that meta-GGA functionals yield a better description of properties of transition metal compounds.^37,38^ However, even at this level of theory, the distinction between Pd^II^–F (1*a*) and Pd^IV^–F (1*b*) distances is still not as pronounced as in the experimentally determined values. The same applies to the magnetic moment of Pd, which is underestimated for Pd^II^ (μ_1*a*_) and overestimated for Pd^IV^ (μ_1*b*_).

Only when employing the hybrid HSE06 functional, DFT calculations yield bond lengths and magnetic moments in close agreement with experiment (Table 1). Moreover, the charges on both Pd sites are clearly differentiated, in accordance with the Pd^II^Pd^IV^F_6_ formulation. Finally, both the ferromagnetic ground state of Pd_2_F_6_ as well as its insulating properties^20^ are well reproduced with this DFT functional. This indicates that using this method is essential for the correct description of the mixed-valent nature of palladium trifluoride. It is also noteworthy that the HSE06 functional gives the enthalpy of formation of Pd_2_F_6_ (−1.36 eV per atom) in close agreement with the experimental value (−1.25 eV per atom).^39^ Therefore, a computationally intensive HSE06 method was chosen to perform modeling of the properties of this compound (including the phonon structure and Raman activity).

Electrical measurements performed on Pd_2_F_6_ compressed to 8 GPa indicated a drastic reduction in resistance upon increase of pressure,^19,20^ which led to a hypothesis that Pd^II^–F and Pd^IV^–F distances equalize upon compression yielding a genuine Pd^III^ compound (Pd^III^F_3_). This interpretation is not supported by the HSE06 calculations, as it is found that at 10 GPa, the two Pd sites in the LiSbF_6_-type polymorph still have differing Pd–F distances (2.11/1.89 Å), magnetic moments (1.56/0.06 μ_B_), and charges (1.4/1.9*e*). However, these calculations predict that upon compression to 10 GPa the band gap of the LiSbF_6_-type structure of Pd_2_F_6_ is reduced by 0.2 eV, which might explain the experimentally observed decrease in resistivity.

**Figure 1:**
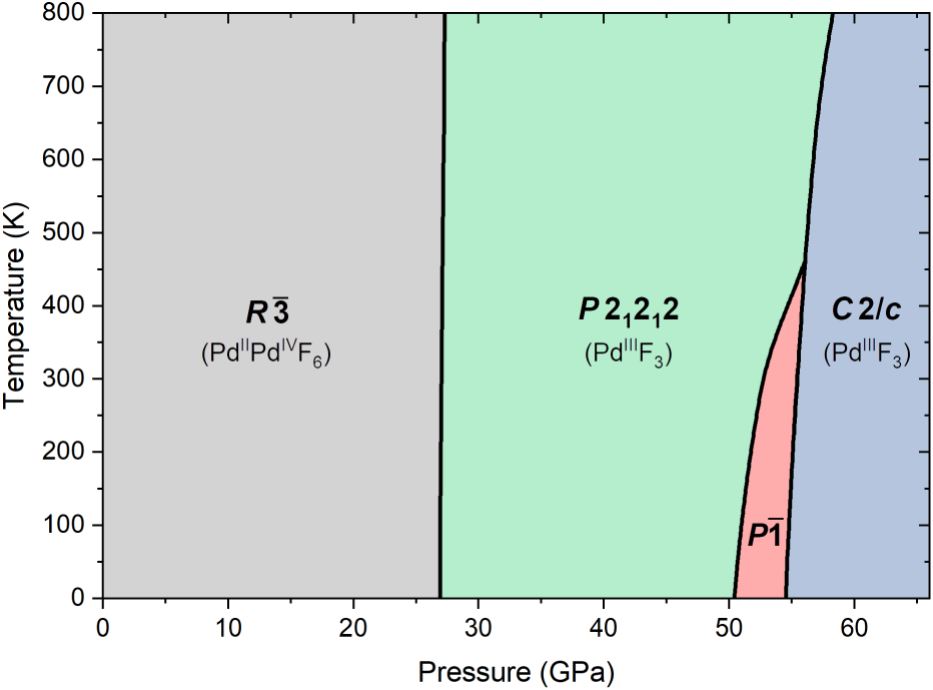
Calculated phase diagram of palladium trifluoride. The black lines mark boundaries between various phases.

The stability of the charge-ordered mixed-valent *R*3̅ structure at modest compression is corroborated by the phase diagram of palladium trifluoride modeled with the quasi-harmonic approximation (QHA) based on HSE06 calculations (Figure 1). The ambient-pressure LiSbF_6_-type structure remains the most stable polymorph of palladium trifluoride up to 27 GPa, in contrast to previous PBE-calculations which predicted a transition into a tetragonal (*P*4_1_2_1_2) metallic polymorph above 19 GPa.^34^ In fact, HSE06 calculations indicate that this tetragonal structure is dynamically unstable, i.e., exhibits imaginary modes in the phonon dispersion curves. Applying the distortion of one of these modes and reoptimizing the structure yields a orthorhombic structure of *P*2_1_2_1_2 symmetry which turns out to be the most stable polymorph up to around 50 GPa (Figure 1). The predicted phase sequence at room temperature and up to 70 GPa is . The triclinic *P*1̅ and monoclinic *C*2/*c* structures have been predicted earlier, but with different stability fields.^34^

**Figure 2:**
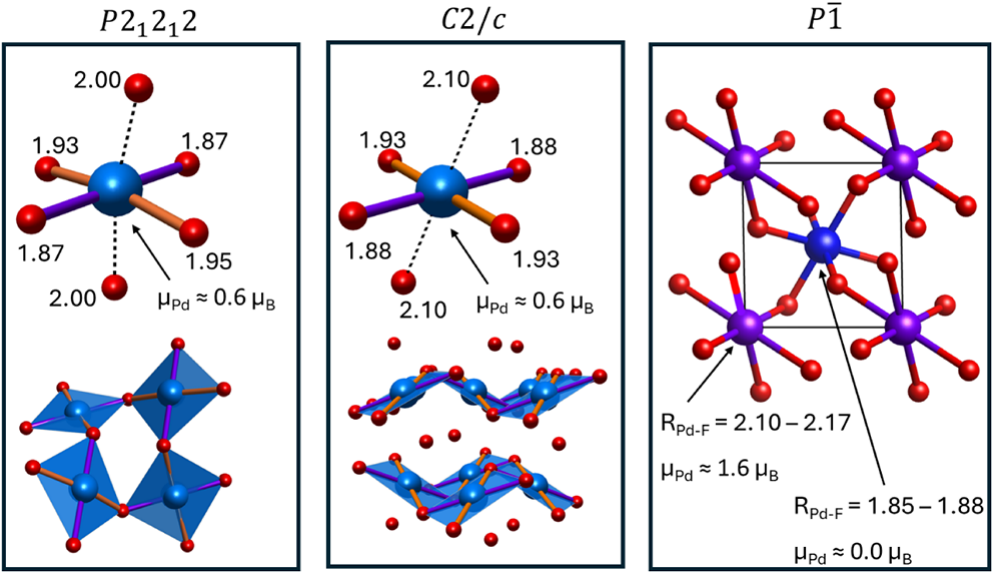
Comparison of the palladium coordination geometry in the *P*2_1_2_1_2, *C*2/*c*, and *P*1̅ polymorphs of PdF_3_ computed at 40 GPa. Pd–F distances are given in Å. Violet, light blue, and dark blue atoms denote Pd^II^, Pd^III^, and Pd^IV^ sites, respectively, whereas red balls mark F atoms.

The analysis of the calculated geometry (see Table S2 for details) and magnetic properties of the *P*2_1_2_1_2 and *C*2/*c* polymorphs clearly indicates that both contain solely Pd^3+^ cations. First, all Pd sites in these phases, which are computed to be insulators, exhibit the same magnetic moment of 0.6 μ_B_ (Table 2), in line with the expected value for a spin-1/2 (4d^7^) Pd^3+^ cation. We note that similar values of the orbital magnetic moment were obtained for related 3d^9^ (La_2_CuO_4_) and 4d^9^ (AgF_2_) spin-1/2 systems.^40−42^ Second, the computed charges are equal for all Pd sites, and have values almost exactly in between that of Pd^2+^ and Pd^4+^ cations in *R*3̅. Finally, in both compounds the coordination sphere of palladium takes shape of an elongated octahedron (Figure 2), as expected for a cation with an uneven occupation of the *e*_*g*_ antibonding orbitals.

**Table 2:** Properties of Palladium Trifluoride Polymorphs Calculated at 40 GPa^a^

		*q*_Pd_		μ_Pd_	
Pd^II^Pd^IV^F_6_	*R*3̅	1.4	1.9	1.53	0.04
	*P*1̅	1.4	1.9	1.59	0.07
Pd^III^F_3_	*P*2_1_2_1_2	1.7		0.65	
	*C*2/*c*	1.7		0.63	

a Mulliken charges of Pd atoms (*q*_Pd_) are given in elementary charge, magnetic moments (μ_Pd_) in μ_B_.

Therefore, both the *P*2_1_2_1_2 and *C*2/*c* polymorph correspond to a single-valent (comproportionated) Pd^III^F_3_ formulation. In contrast, the *P*1̅ polymorph exhibits characteristics of a mixed-valent (disproportionated) compound (Pd^II^Pd^IV^F_6_) with an uneven distribution of charges and magnetic moments between Pd atoms, resembling that found in the *R*3̅ structure (Table 2). The general picture emerging from the calculations is a preference for a single Pd^III^ valency in palladium trifluoride for *P* > 30 GPa, with a very narrow region of stability for the mixed-valent Pd^II^/Pd^IV^*P*1̅ polymorph appearing at low temperatures (*T* < 300 K) between 51 and 54 GPa.

### High-Pressure Experiments

**Figure 3:**
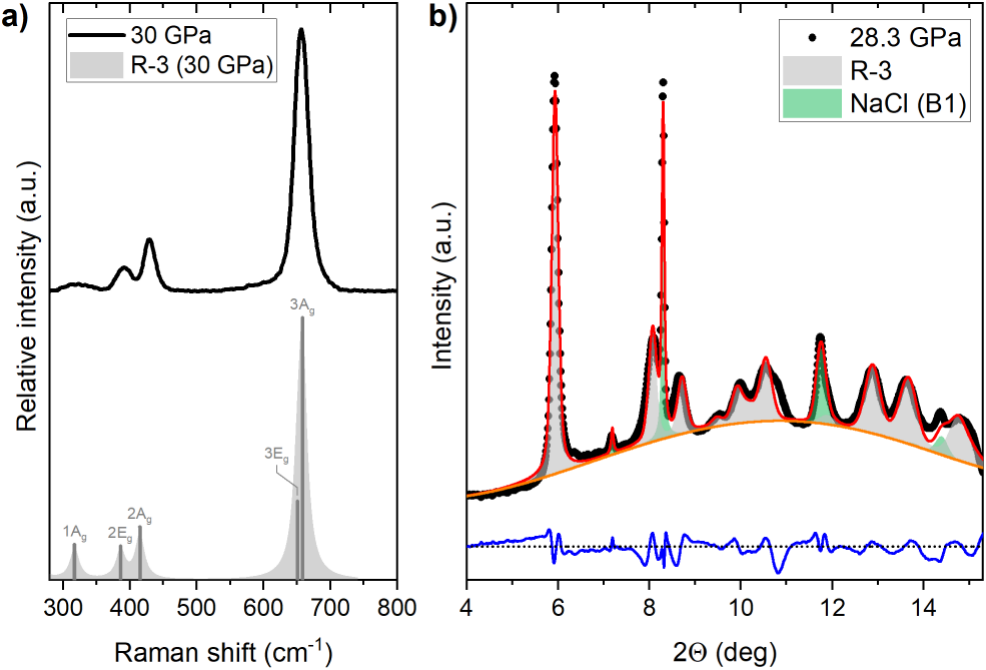
(a) Raman spectrum of powdered palladium trifluoride at 30 GPa (black line) together with that modeled by DFT for the *R*3̅ structure at the same pressure (gray lines). The labels indicate the irreproducible representation of the Raman-active bands, with those of the same symmetry numbered according to their increasing frequency. (b) LeBail fit of the PXRD data (black dots) obtained at 28.3 GPa. Red lines indicate the profile derived from the model incorporating the *R*3̅ polymorph (gray Bragg peaks) and the NaCl pressure marker (green peaks). Blue line gives the difference between model and experiment. The refinement *R*_wp_ factor is 2.43%.

**Figure 4:**
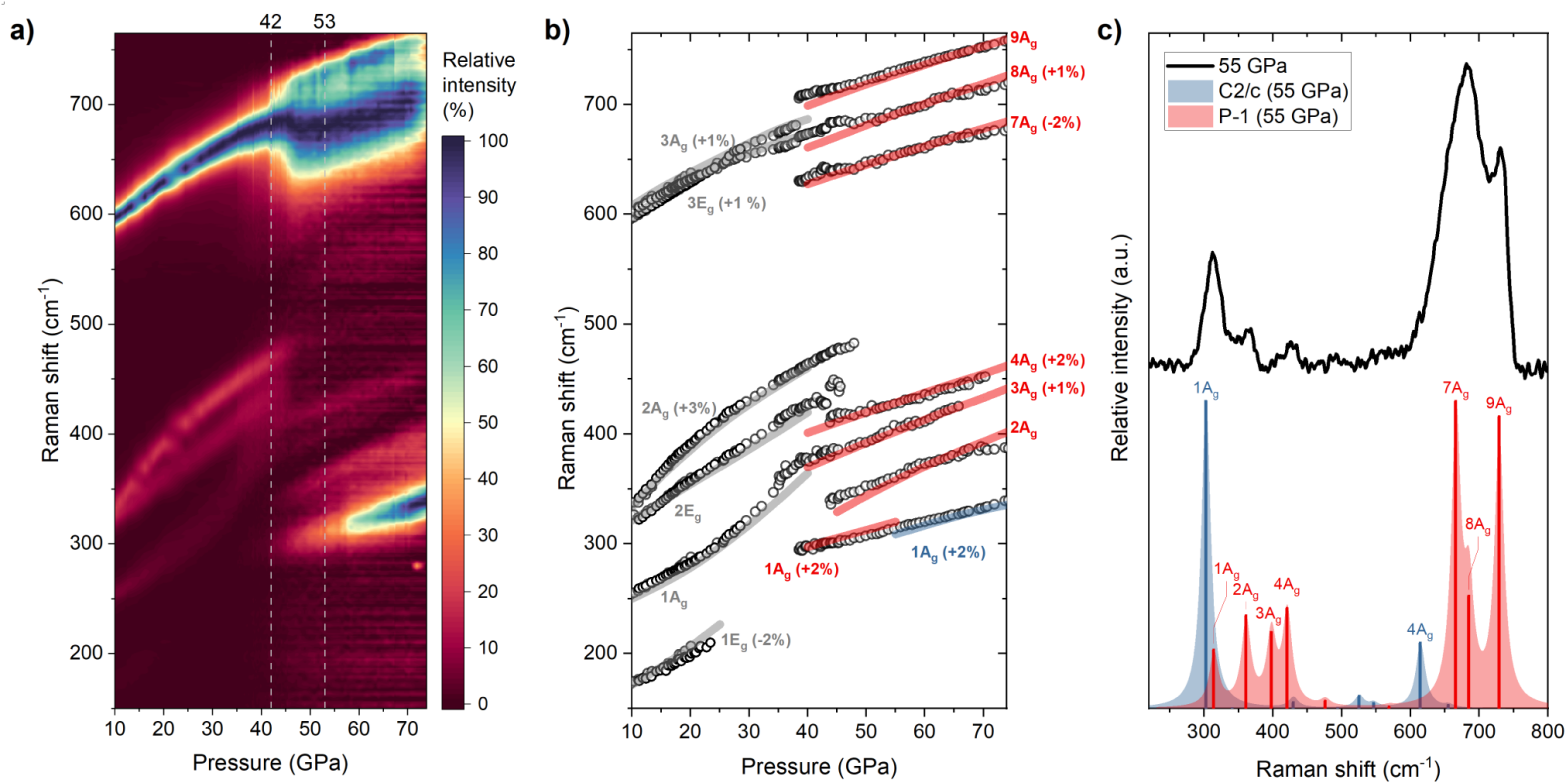
(a) Heat map depicting the evolution of the Raman spectrum of palladium trifluoride upon compression. (b) Pressure dependence of the position of Raman bands: experimental data are given by black circles while the DFT-derived positions of the most intense bands of the *R*3̅, *P*1̅, and *C*2/*c* phases are shown by gray, red, and blue lines, respectively. For clarity the frequencies of some modes were scaled, as indicated. (c) Comparison of the experimental (black line) and theoretical (red lines—*P*1̅, blue lines—*C*2/*c*) Raman spectra at 55 GPa. In (b) and (c) vibrational modes are labeled by their irreproducible representation, those of the same symmetry are numbered according to their increasing frequency.

In an attempt to verify this prediction experimentally, high-pressure diamond anvil cell (DAC) experiments have been performed on solid palladium trifluoride, measuring in situ Raman scattering and powder X-ray diffraction of this compound compressed up to pressures of 74 and 59 GPa, respectively. Both techniques indicate that the disproportionated LiSbF_6_-type polymorph is stable at pressures exceeding that of the previous experiment (8 GPa).^19,20^ The Raman spectrum collected at 30 GPa agrees well with that obtained by DFT calculations for this phase (Figure 3a), and the PXRD pattern recorded at 28 GPa can be modeled assuming that palladium trifluoride exhibits the *R*3̅ structure (Figure 3b). Importantly, the unit-cell volume obtained through a LeBail fit at this pressure is within 1.4% of that obtained by hybrid DFT modeling. Moreover, below 30 GPa we observe spectral features originating from all of the six Raman-active modes (3·A_g_ + 3·E_g_) of the LiSbF_6_-type structure, with only the weakest band, stemming from the 1E_g_ mode, disappearing above 24 GPa (Figure 4).

The 1A_g_, 2A_g_, 2E_g_ modes form three distinct bands visible below 500 cm^–1^, while the two high-frequency modes (3E_g_, 3A_g_) form components of a single, and strong, peak located above 600 cm^–1^ (Figure 3a). Importantly, the pressure dependence of the frequencies of all modes is well reproduced by DFT modeling performed for the LiSbF_6_-type polymorph (Figure 4b).

In contrast to DFT predictions, upon compression above 30 GPa we do not witness a transition from the *R*3̅ polymorph into the *P*2_1_2_1_2 structure. Instead, the Raman scattering signature of the *R*3̅ polymorph is observed up to 42 GPa. Only above this pressure we observe indications of a phase change, as the bands originating from 1A_g_, 2A_g_, and 1E_g_ modes disappear and the high-frequency peak broadens into a band that can be described by three components (Figure 4a,b). This is accompanied by the appearance of four new Raman bands below 500 cm^–1^, with the intensity of the lowest-frequency one (located at 300 cm^–1^ at 42 GPa) rapidly growing above 53 GPa. At these pressures, this band is assigned to the strongest Raman-active mode 1A_g_ of the *C*2/*c* polymorph (Figure 4c), and its emergence interpreted as the sign of palladium trifluoride starting to transition to this structure above 53 GPa. The transition is slow, as the 1A_g_ peak becomes the dominant Raman band only above 71 GPa.

The Raman signature of the phase appearing above 42 GPa, which sluggishly transforms to *C*2/*c* above 53 GPa, is matched by that modeled for the *P*1̅ polymorph—both in terms of band position and intensity (Figure 4b,c). Therefore, the picture emerging from Raman scattering measurements is that of the *R*3̅ polymorph transforming abruptly into the *P*1̅ structure at 42 GPa, followed by a sluggish *P*1̅ → *C*2/*c* phase transformation starting at 53 GPa. All of the three polymorphs are also observed during decompression: the characteristic single-band Raman spectrum of *C*2/*c* persists down to 43 GPa, and is abruptly replaced at this pressure by the spectrum associated with *P*1̅, which in turns transforms to that of the starting *R*3̅ phase below 18 GPa (Figure S2).

**Figure 5:**
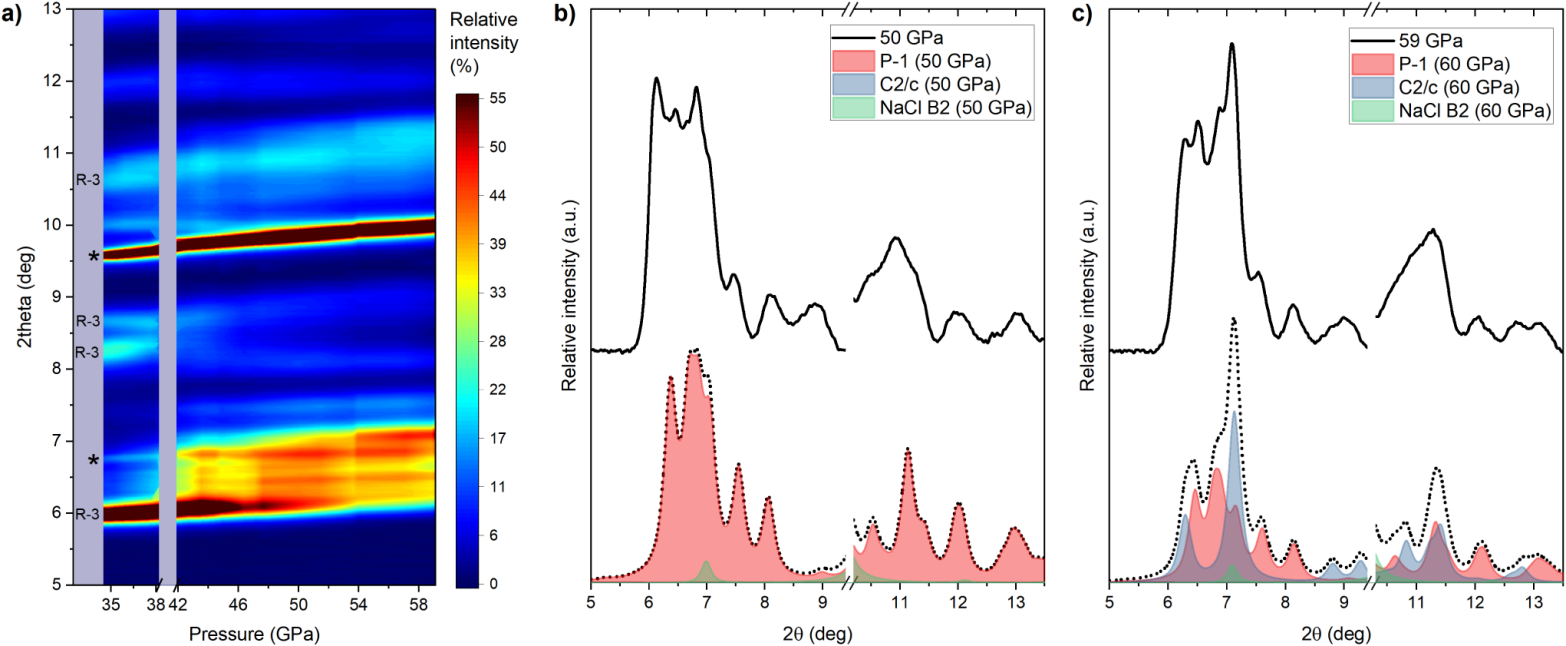
(a) Color map depicting the evolution of the PXRD diffractograms of palladium trifluoride upon compression. Most intense Bragg peaks of the *R*3̅ phase are highlighted together with those originating from the B2 (CsCl) structure of NaCl (marked with an asterisk). PXRD diffractograms collected at (b) 50 GPa and (c) 59 GPa and simulated PXRD patterns for DFT-optimized structures of the *P*1̅ and *C*2/*c* polymorphs of palladium trifluoride (red and blue, respectively, a 2:3 molar ration between *P*1̅ and *C*2/*c* was assumed in (c)). These diffractograms are summed together with that corresponding to B2 NaCl (green), following its equation of state,^43^ to give the simulated dotted black curve. In (b) and (c) the 2θ range in which the strong (110) peak of NaCl is present (9.4–10.4°) has been omitted.

The results of Raman measurements are corroborated by PXRD data in which the disappearance of reflections associated with the LiSbF_6_ polymorph above 42 GPa is observed (Figure 5a). Unfortunately, the broadening of Bragg peaks upon the phase transition, along with the coexistence and low symmetry of the *P*1̅ and *C*2/*c* phases, hindered attempts of obtaining meaningful Rietveld refinements of the experimental data above 42 GPa. However, the PXRD pattern collected at 50 GPa agrees reasonably well with that calculated for the DFT-optimized crystal structure of the *P*1̅ phase (Figure 5b). Moreover, the evolution of the diffractograms at higher pressures, most notably the appearance of a strong Bragg peak at 2θ = 7.1° is consistent with the formation of the *C*2/*c* phase, which becomes the main component of the two-polymorph mixture at 59 GPa, as shown in Figure 5c (see also Figure S3). Therefore, both Raman scattering and powder X-ray diffraction measurements indicate that the ambient-pressure *R*3̅ phase transforms to the *P*1̅ structure above 42 GPa, followed by the gradual formation of the *C*2/*c* phase at pressures exceeding ∼50 GPa.

Room-temperature DAC experiments do not indicate the presence of the *P*2_1_2_1_2 phase at any pressure below 74 GPa, in contrast to DFT modeling which predicts it to be the most stable polymorph between 27 and 53 GPa (Figure 1). This discrepancy is most probably due to large energetic barriers associated with the *R*3̅ → *P*2_1_2_1_2 transition which kinetically hinder its formation. In fact, if one assumes that the *P*2_1_2_1_2 is inaccessible due to slow kinetics of its formation, DFT-derived Gibbs free energies (Figure S4) yield the following phase transition sequence at 300 K: , in close agreement with the one observed in the DAC experiment (). The persistence of some polymorphs beyond the region of their thermodynamic stability, due to large kinetic barriers associated with the phase transitions into thermodynamically favored phases, has also been observed for other fluorides and oxides.^18,44,45^

**Figure 6:**
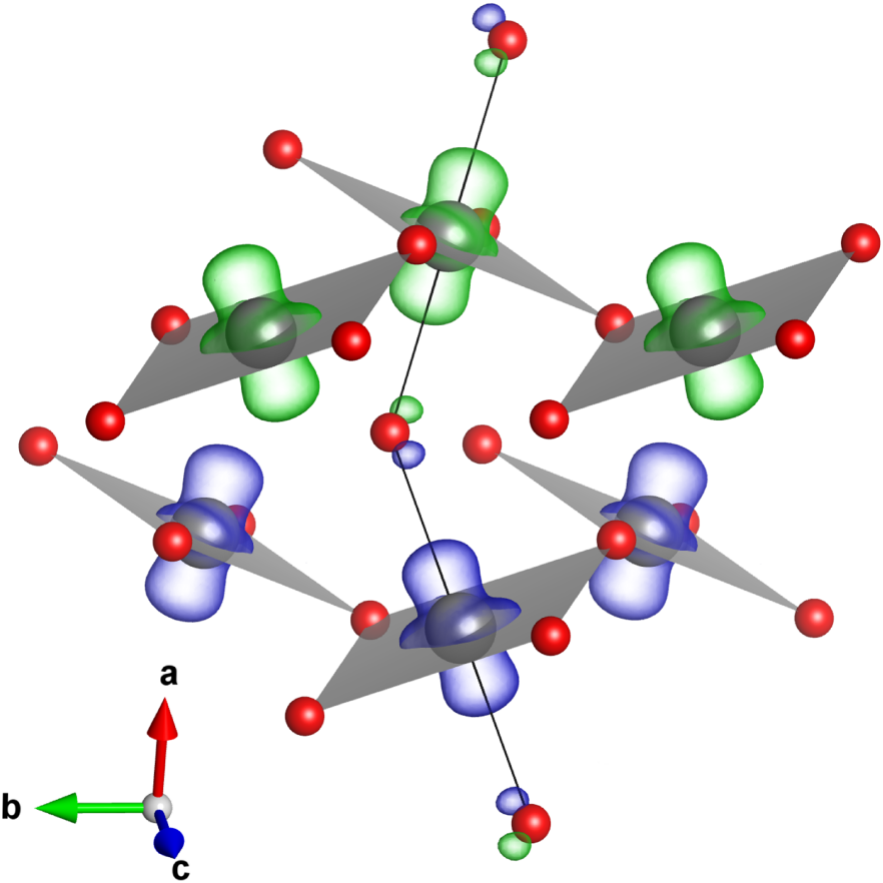
Structure of the *C*2/*c* phase of palladium trifluoride (gray and red balls mark Pd and F atoms, respectively). Four shorter Pd–F distances (DFT values at 60 GPa: 2 × 1.87 Å + 2 × 1.91 Å) form a distorted square around Pd (marked with a gray plaquette). The longer Pd–F contacts (2.08 Å) are shown with black lines. Spin-density isosurfaces (drawn at 0.24 Å^–3^) are shown in green/blue for up/down spin density.

To the best of our knowledge, both the *P*1̅ and *C*2/*c* phases of palladium trifluoride constitute novel structure types, despite being isosymmetric with CuMoF_6_,^46^ and the ambient-pressure structure of MnF_3_,^47^ respectively. The *C*2/*c* structure is also distinct from the YF_3_-type structure (*Pnma*) adopted by FeF_3_ and MnF_3_ at high pressure.^10,11^ Additional insights into this phase can be gained from the analysis of the geometry and spin density of the structure obtained from DFT. The four shorter Pd–F contacts of the elongated distorted octahedral coordination sphere of palladium form plaquettes joined into a corrugated sheets of [PdF_4/2_]^+^ stoichiometry which are separated by fluorine anions lying in the (100) plane (Figure 6). These anions form two longer Pd–F contacts completing the 6-fold Pd coordination sphere.

Despite the two-dimensional (2D) character of the Pd–F substructure, the spin density has a one-dimensional (1D) distribution, as can be seen in Figure 6. The unpaired electrons reside on a d_*z*^2^_-like orbital contained in the direction of the longer Pd–F contacts, i.e., the direction of the local *z* axis of the elongated octahedron, in accordance with what is expected for a d^7^ ion. The strong overlap of d_*z*^2^_-like orbitals of Pd^3+^ cations from successive planes, and the weak interaction of cations within the same plane, might lead to strong 1D superexchange interactions, as found in other spin-1/2 systems.^48−52^ While a detailed investigation of the strength of various magnetic coupling paths in *C*2/*c* PdF_3_ is beyond the scope of this study, we note that the difference in DFT-derived energies between the lowest spin state of *C*2/*c*, characterized by antiferromagnetic (AFM) coupling between [PdF_4/2_]^+^ layers (Figure 6), and that of a ferromagnetic solution reaches 135 meV per PdF_3_ at 70 GPa. Such a large difference, equivalent to temperatures of 1500 K, hints at very strong interlayer AFM coupling.

## Conclusions

Through a combination of high-level hybrid DFT calculations and DAC experiments we unveil the high-pressure behavior of palladium trifluoride. We show that the computationally intensive hybrid DFT method is essential for the correct description of the mixed-valent (disproportionated) nature (Pd^II^Pd^IV^F_6_) of this compound. The DFT-derived phase diagram indicates the prevalence of comproportionated (Pd^III^F_3_) phases, of *P*2_1_2_1_2 and *C*2/*c* symmetry, at pressures above 27 GPa. In room-temperature DAC experiments, we do not find evidence of formation of the orthorhombic phase, most likely due to the large kinetic barriers associated with its formation. However, both Raman scattering and PXRD *in situ* measurements reveal two other phase transitions: the transformation from the ambient-pressure *R*3̅ structure (LiSbF_6_-type) into a triclinic phase (*P*1̅) at 42 GPa followed by a sluggish *P*1̅ → *C*2/*c* transition commencing at about 50 GPa. While palladium trifluoride retains its mixed-valence nature (Pd^II^Pd^IV^F_6_) in the first two phases, the monoclinic polymorph is a genuine Pd^III^F_3_ system. Therefore, pressure can drive comproportionation of palladium trifluoride, but this requires greater pressures than previously suggested (50 GPa instead of 8 GPa),^19,20^ as well as a phase change from the structure observed at ambient conditions. Although the ambient-pressure structure of palladium trifluoride (*R*3̅, LiSbF_6_-type) is closely related to that of FeF_3_ and MnF_3_ (*R*3̅*c*, VF_3_-type), both the modeled and observed phase transition sequence reported here greatly differ from that of these two lighter fluorides, which both transform to a YF_3_-type polymorph (*Pnma*) upon compression.^10,11^ In fact all of the three high-pressure polymorphs of palladium trifluoride (*P*2_1_2_1_2, *P*1̅, *C*2/*c*) studied here seem to be novel structure types, not observed in other systems. Whether these structures are unique for the trifluoride of palladium, or might be observed also in other compounds, such as nickel trifluoride or CuPdF_6_, remains an open question. Another interesting point for future investigations are the magnetic properties of the *C*2/*c* phase. The analysis of the geometry of the superexchange paths in this system, as well as preliminary DFT results, suggest the possibility of very strong and highly anisotropic AFM coupling developing in this phase.

## Experimental and Computational Methods

Palladium trifluoride was synthesized by the reaction of elemental fluorine with palladium, following synthetic procedures described in previous studies.^20,25,28^ Details of the synthesis and sample characterization at ambient conditions are given in the Supporting Information (Figure S1).

Five high pressure runs were performed with piston–cylinder diamond anvil cells (SymmDAC from Almax easyLab bv, and iBX-80 from DAC Tools LLC) fitted with pairs of low-fluorescence diamonds with culet diameters ranging from 200 to 400 μm. Powdered palladium trifluoride was enclosed in a hole (60 to 150 μm in diameter) made by spark erosion in a stainless-steel gasket preindented to a thickness of 25 to 35 μm. In four runs, the sample was loaded neat into the DAC, whereas in one experiment NaCl was used as a pressure-transmitting medium (PTM)^53^ (Figure S5). The results obtained in the runs with and without the PTM were consistent. In Raman scattering experiments pressure was determined from the shift of the first-order Raman signal of the diamond anvil.^54^ The spectra were collected at room temperature using an Alpha300M+ confocal microscope (Witec)—for details see Supporting Information. The positions of the Raman bands were determined using the Fityk 1.3.1 software,^55^ by fitting the observed bands with pseudo-Voigt profiles. To confirm sample homogeneity, area scans encompassing the whole sample chamber were conducted at selected pressures. High-pressure powder X-ray diffraction measurements were performed at the DanMAX beamline at the MAX IV synchrotron (λ = 0.3542 Å, beam size ∼19 × 15 μm^2^ with NaCl acting both as the PTM and a pressure gauge.^43^ The diffractograms were recorded on a Dectris Pilatus3 X 2M CdTe area detector calibrated with a Si standard. All 2D diffractograms were integrated using the Dioptas software,^56^ and subsequent LeBail fit was performed using MAUD.^57^ During the refinement, the atomic positions were fixed at values derived from the DFT calculations.

Solid-state spin-polarized density functional theory (DFT) calculations of the geometry and enthalpy of the high-pressure polymorphs of palladium trifluoride were performed with the use of the HSE06 functional,^36^ as implemented in the plane-wave VASP 6.4.3 code.^58−60^ The cutoff energy of the plane waves was set to 700 eV with a self-consistent field convergence criterion of 10^–7^ eV. The *k*-point mesh was set at 2π × 0.05 Å^–1^. All structures were optimized using a conjugate gradient algorithm until forces acting on the atoms were less than 5 meV/Å (for more details see Table S1). Geometry optimization was carried out for the lowest energy spin state. This method yielded lattice constants and Pd–F distances of the LiSbF_6_-type structure of Pd_2_F_6_ within 2% of those determined experimentally at ambient pressure.^20^ The phonon dispersion curves and Raman scattering intensities were calculated using the finite-displacement supercell approach as implemented in the PHONOPY and Phonopy-spectroscopy codes.^61,62^ The *P*–*T* phase diagram was constructed by comparing Gibbs free energies computed for the various polymorphs under finite temperature conditions using the quasi-harmonic approximation (QHA) implemented in the PHONOPY-QHA script^63^ using the Vinet equation of state (EoS).^64^ Mulliken gross orbital populations and charges^65^ were calculated through the Lobster code (version 5.1.1)^66^ by projecting the DFT plane-wave functions onto a localized basis set.

## Data Availability

The data supporting the findings of this study are available on the Repository for Open Data server (https://repod.icm.edu.pl/) under the DOI number: 10.18150/SG2SSB.

## Supporting Information Available

Details of the Pd_2_F_6_ synthesis and Raman setup, the room-temperature PXRD diffractogram of Pd_2_F_6_ synthesized in this study, input files for VASP calculations, structural details of studied phases and comparison of their geometry, Raman spectra upon decompression, evolution of the PXRD diffractograms of palladium trifluoride between 48 and 59 GPa, computed relative Gibbs free energies (PDF)
