# Supplementary material for: Pressure-Induced Comproportionation in Palladium Trifluoride
Source: Inorg Chem. 2025 Apr 30;64(18):9026–34. doi: 10.1021/acs.inorgchem.5c00465 (PMC12076551; doi:10.1021/acs.inorgchem.5c00465)
Supplement: Supplementary file 1 — ic5c00465_si_001.pdf [file ic5c00465_si_001.pdf]

SUPPORTING INFORMATION

Pressure-Induced Comproportionation in Palladium Trifluoride

Sylwia Olszewska,[1] Sharad Babu Pillai,[1] Deepak Upadhyay,[1] Kinga Zdun,[1] Jakub Drapała,[2] Klemen Motaln,[3,4] Mirela Dragomir,[3,4] Matic Lozinšek,[3,4]* and Dominik Kurzydłowski[1]*

[1] *Faculty of Mathematics and Natural Sciences, Cardinal Stefan Wyszynski University in Warsaw, Warsaw 01-938, Poland*

[2] *Faculty of Chemistry, Warsaw University of Technology, Warsaw 00-664, Poland*

[3] *Jožef Stefan Institute, Ljubljana 1000, Slovenia*

[4] *Jožef Stefan International Postgraduate School, Ljubljana 1000, Slovenia*

* *matic.lozinsek@ijs.si, d.kurzydlowski@uksw.edu.pl*

Synthesis of Pd_2F_6

In a glovebox with argon atmosphere and controlled water content (below 1 ppm), 160 mg of Pd powder (its purity was checked by PXRD) was added to a PTFE-gasketed nickel reaction vessel which has been previously passivated with elemental fluorine. The vessel was removed from the glovebox and connected to a custom-built vacuum line constructed from nickel, copper, polytetrafluoroethylene (PTFE) and fluorinated ethylene propylene (FEP). After evacuation of the vessel, gaseous fluorine (800 Torr pressure) was dosed into the reactor at room temperature. The vessel was then placed in a small tube furnace controlled by a Digi-Sense temperature controller. To prevent damage to the PTFE seals, the upper part of the reactor was cooled by circulating cooling water through a cooling ring welded onto the reactor. The furnace was heated to 500 °C and this temperature was maintained for 8 hours; then the heating was turned off and the reactor was left to cool overnight. The next day, the reactor was connected to the vacuum line and the remaining F_2 was removed. The reactor was then returned to the glovebox, where the product was homogenized with a pestle and mortar. Four such heating cycles were performed, with the amount of fluorine consumed decreasing after each cycle.

After the last run, the resulting gray-black powder was filled into a quartz glass capillary (passivated with F_2), which was heat-sealed. PXRD measurements were conducted in transmission mode on a Rigaku OD XtaLAB Synergy-S, Dualflex diffractometer, equipped with a Dectris Eiger2 R CdTe 1M hybrid pixel array detector, using microfocus Ag Kα radiation (λ = 0.56087 Å, 65 kV, 0.67 mA). The powder patterns were processed and extracted by *CrysAlis^{Pro}* software.[S1] Indexing and Rietveld refinements analyses were performed using *GSAS-II* software.[S2] The analysis of the data (Figure S1) showed that the sample contained Pd_2F_6 with the $R\bar{3}$ structure.

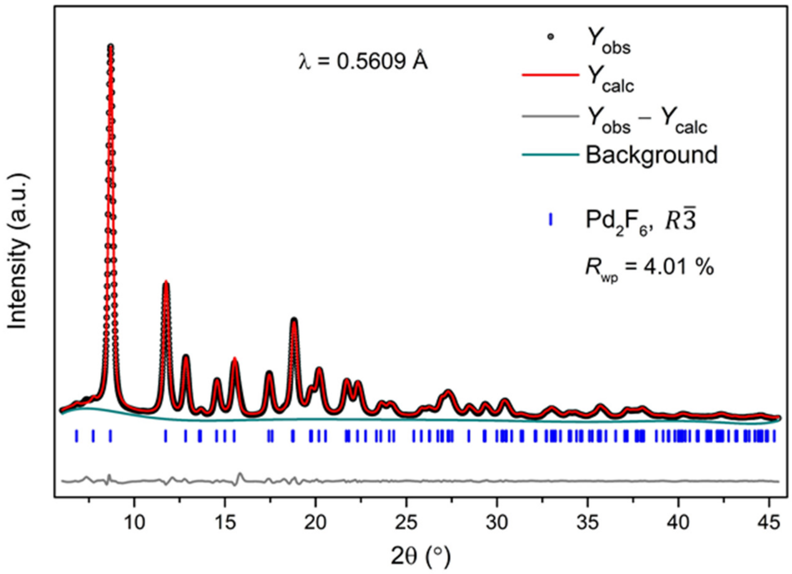

Figure S1. Rietveld refinement of room-temperature PXRD data (black circles) collected with Ag Kα radiation (λ = 0.56087 Å) and the refined profile (red line) for the synthesized Pd_2F_6. The fit incorporates the $R\bar{3}$ structure of Pd_2F_6 (Bragg peaks marked with blue ticks). Green and grey lines mark the background and difference between the model and experimental data, respectively. The R_{wp} factor is 4.01%, the R_{exp} is 1.62%.

Raman measurements

Raman spectra were measured on a WITec Alpha300M+ Raman imaging microscope. A 532 or 633 nm laser line was delivered to the microscope via a single-mode optical fiber, with the laser power at the sample not exceeding 25 mW. The Raman signal was collected through a 50× long working distance objective (NA = 0.40) and transmitted through a photonic crystal fiber to a lens-based spectrometer (Witec UHTS 300, f/4 aperture, focal length 300 mm) equipped with a back-illuminated Andor iDUS 401 detector, which was thermoelectrically cooled to −60 °C. The spectra were acquired using an 1800 mm^{-1} grating, resulting in a spectral resolution of 1.2 cm^{-1} or better. Typical acquisition was done from a 12 × 12 μm^2 area with a 6 × 6 sampling (2 µm spatial resolution). The 36 resulting spectra obtained from the area scan were postprocessed using the *Project FIVE* software (Witec) by performing background subtraction and cosmic ray removal and then averaged into a single spectrum.

DFT calculations

Table S1. Selected keywords used in input files for VASP calculations

INCAR		KPOINTS	POTCAR
PREC = Accurate		Automatic mesh	TITEL = PAW_PBE Pd 04Jan2005
ENCUT = 700		0	TITEL = PAW_PBE F 08Apr2002
IBRION = 2	ISIF = 3	Auto	
EDIFF = 1.00E-07	EDIFFG = -0.005	20	
LHFCALC = .TRUE.	HFSCREEN = 0.2		
ALGO = All	LSUBROT = .TRUE.		
PRECFOCK = Fast	NKRED = 1		
LASPH = .TRUE.			
TIME = 0.3			
VOSKOWN = 1			
LMAXMIX = 4			
ISPIN = 2			
ISMEAR = 0	SIGMA = 0.1		
LORBIT = 14			

Table S2. Structural details of PdF_3 phases calculated at T = 0 K with the HSE06 functional

Structure	Pressure	Lattice parameters	Atomic coordinates
$Pd^{II}Pd^{IV}F_6$<br>$R\bar{3}$	0.001 GPa	a = 5.546 Å<br>α = 54.93°	Pd 1a 0.00000 0.00000 0.00000<br>Pd 1b 0.50000 0.50000 0.50000<br>F 6f 0.27137 0.88026 –0.41576
$Pd^{III}F_3$<br>$P2_12_12$	35 GPa	a = 6.320 Å<br>b = 6.317 Å<br>c = 7.425 Å	Pd 4c 0.25132 0.25240 0.84700<br>Pd 4c 0.25181 0.25147 0.34694<br>F 4c 0.31781 0.54284 0.84013<br>F 4c 0.81372 0.03509 0.85004<br>F 4c 0.03588 0.68381 0.34930<br>F 4c 0.54204 0.18504 0.34095<br>F 4c 0.31500 0.49428 0.49682<br>F 4c 0.00844 0.68541 0.00114
$Pd^{II}Pd^{IV}F_6$<br>$P\bar{1}$	55 GPa	a = 3.975 Å<br>b = 4.136 Å<br>c = 4.227 Å<br>α = 90.39°<br>β = 96.49°<br>γ = 103.15°	Pd 1a 0.0000 0.00000 0.00000<br>Pd 1h 0.50000 0.50000 0.50000<br>F 2i 0.26727 0.08452 0.60193<br>F 2i 0.49370 0.73175 0.87527<br>F 2i 0.89513 0.41802 0.73748
$Pd^{III}F_3$<br>$C2/c$	60 GPa	a = 6.459 Å<br>b = 4.380 Å<br>c = 4.616 Å<br>β = 90.11°	Pd 4c 0.25000 0.25000 0.00000<br>F 8f 0.35711 0.38069 0.63494<br>F 4e 0.00000 0.60414 0.25000

DAC measurements

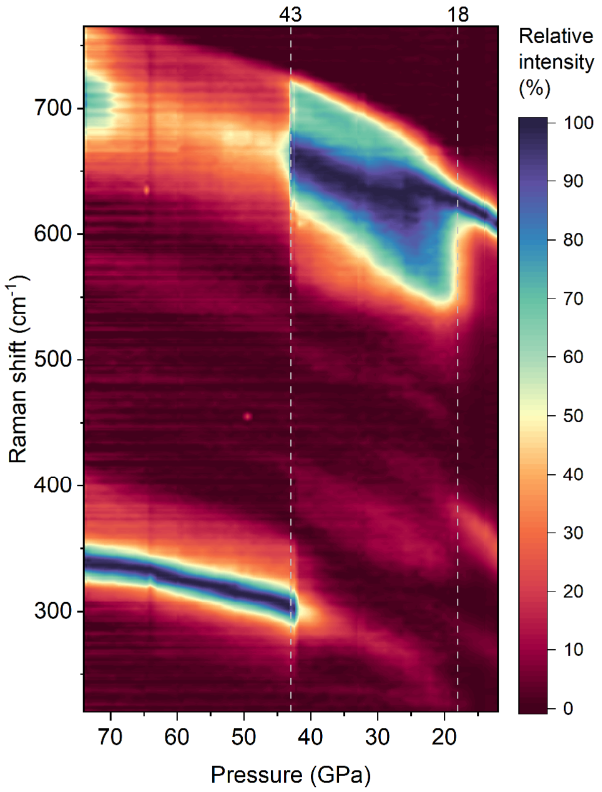

Figure S2. Color map depicting the evolution of the Raman spectrum of palladium trifluoride upon decompression.

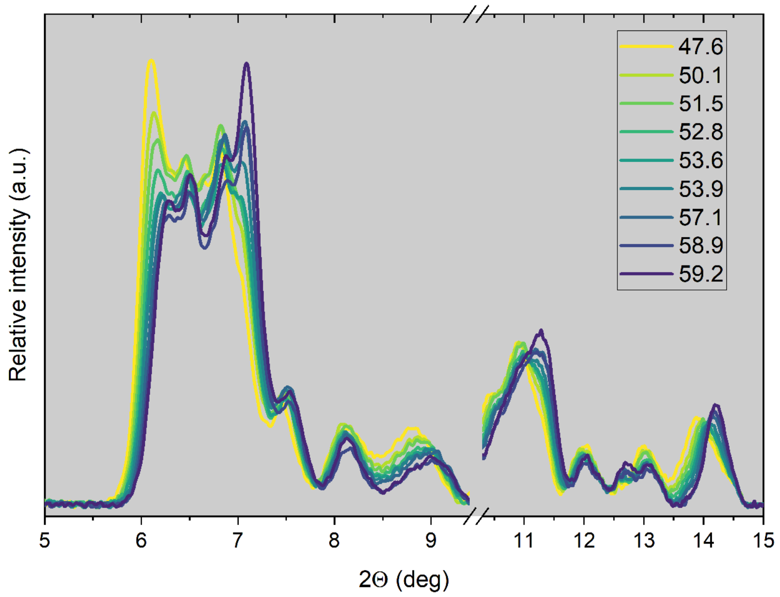

Figure S3. Evolution of the PXRD diffractograms of palladium trifluoride between 48 and 59 GPa. The 2θ range in which the strong 110 reflection of NaCl is present (9.4–10.4°) has been omitted.

QHA calculations

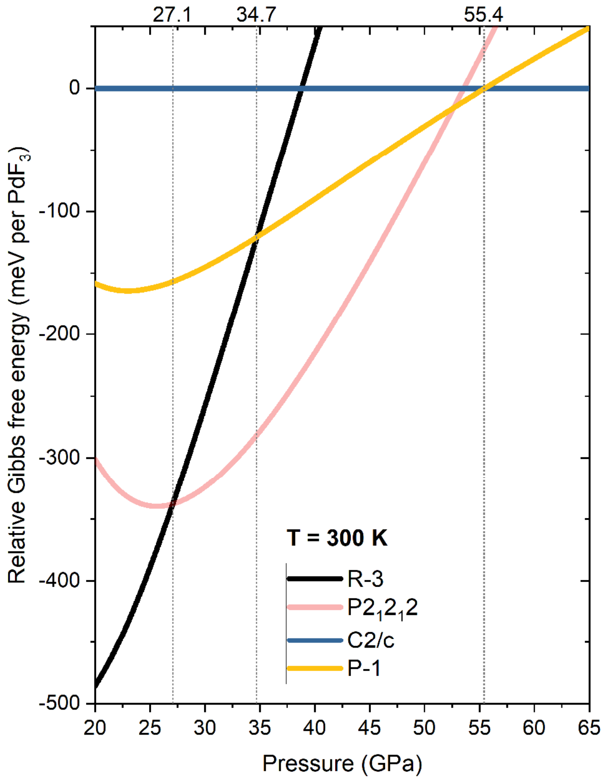

Figure S4. Computed relative Gibbs free energy (referenced to that of the $C2/c$ structure) of palladium trifluoride at 300 K. The pressures of the $R\bar{3} \rightarrow P2_12_12$, $R\bar{3} \rightarrow P\bar{1}$, and $P\bar{1} \rightarrow C2/c$ phase transitions are given in GPa.

DAC chamber

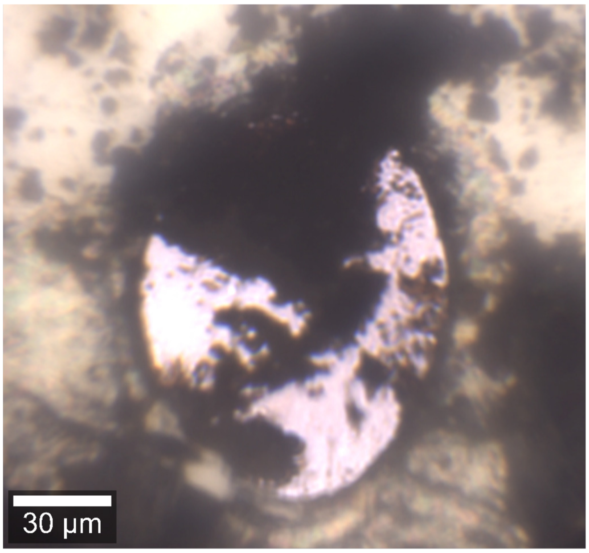

Figure S5. View of the DAC sample chamber in the run with NaCl used as the pressure-transmitting medium. Black powder – PdF_3; translucent powder – NaCl; yellowish grey area – stainless steel gasket.

References

[S1] Rigaku OD. *CrysAlis^{Pro}*. Rigaku Corporation, Wrocław, Poland, **2023**.

[S2] Toby, B. H.; Von Dreele, R. B. *GSAS-II*: the genesis of a modern open-source all purpose crystallography software package. *J. Appl. Crystallogr.* **2013**, *46*, 544–549.
DOI: 10.1107/S0021889813003531
